# Insufficient compensatory pancreatic *β*-cells function might be closely associated with hyperuricemia in U.S. adults: evidence from the National Health and Nutrition Examination Survey

**DOI:** 10.1186/s12889-023-17471-0

**Published:** 2024-01-03

**Authors:** Tianran Shen, Qiutong Zheng, Liling Zhong, Xia Zeng, Xiaojing Yuan, Fengxin Mo, Shiheng Zhu, Wenhan Yang, Qingsong Chen

**Affiliations:** 1 Department of Nutrition and Food Hygiene, School of Public Health, Guangdong Pharmaceutical University, Guangzhou, Guangdong Province 510006 China; 2 Guangdong Provincial Engineering Research Center of Public Health Detection and Assessment, Guangdong Pharmaceutical University, Guangzhou, Guangdong Province 510006 China; 3 Department of Child and Adolescent Health, School of Public Health, Guangdong Pharmaceutical University, Guangzhou, Guangdong Province 510006 China; 4 Department of Occupational Health, School of Public Health, Guangdong Pharmaceutical University, Guangzhou, Guangzhou Province 510006 China

**Keywords:** Hyperuricemia, Pancreatic function, HOMA, NHANES, *β*-cells compensation

## Abstract

**Background:**

The prevalence of hyperuricemia (HUA) is gradually increasing worldwide. HUA is closely related to diabetes, but the relationship between HUA and pancreatic *β*-cells function in the population is unclear. The purpose of this article is to investigate the association between pancreatic *β*-cells and HUA.

**Methods:**

This cross-sectional study examined the association between pancreatic *β*-cells and HUA in 1999–2004 using data from the National Health and Nutrition Examination Survey (NHANES). Subjects were divided into two groups: HUA and non-HUA. Pancreatic *β*-cells function levels were assessed using homeostasis model assessment version 2-%S (HOMA2-%S), homeostasis model assessment version 2-%B (HOMA2-%B) and disposition index (DI). Multivariate logistic regression models and restricted cubic spline models were fitted to assess the association of pancreatic *β*-cells function with HUA.

**Results:**

The final analysis included 5496 subjects with a mean age of 46.3 years (standard error (SE), 0.4). The weighted means of HOMA2-%B, HOMA2-%S and DI were 118.1 (SE, 1.0), 69.9(SE, 1.1) and 73.9 (SE, 0.7), respectively. After adjustment for major confounders, participants in the highest quartile of HOMA2-%B had a higher risk of HUA (O*R* = 2.55, 95% CI: 1.89–3.43) compared to participants in the lowest quartile. In contrast, participants in the lowest quartile of HOMA2-%S were significantly more likely to have HUA than that in the highest quartile (O*R* = 3.87, 95% CI: 2.74–5.45), and similar results were observed in DI (O*R* = 1.98, 95% CI: 1.32–2.97). Multivariate adjusted restricted cubic spline analysis found evidence of non-linear associations between HOMA2-%B, HOAM2-%S, DI and the prevalence of HUA.

**Conclusion:**

Our finding illustrated the indicators of inadequate *β*-cells compensation might be a new predictor for the presence of HUA in U.S. adults, highlighting a critical role of pancreatic *β*-cells function on HUA.

**Supplementary Information:**

The online version contains supplementary material available at 10.1186/s12889-023-17471-0.

## Introduction

Uric acid is produced during purine metabolism [1], and abnormally high serum uric acid (SUA) concentrations can lead to hyperuricemia(HUA) [2]. HUA is a common chronic disease with a progressive increase in prevalence worldwide and a relatively high prevalence in developed countries [3]. Epidemiological evidence suggests that the prevalence of HUA in the United States is as high as 20.1% (estimated 47.13 million people) [4]. HUA is an independent risk factor for several diseases, including hypertension [5], type 2 diabetes [6], nonalcoholic fatty liver disease [7], and cardiovascular disease [8].

*β*-cells are the cells in the pancreas that produce and release insulin in response to blood glucose levels. Insulin helps control blood sugar or glucose levels in the body. When blood sugar rises, *β*-cells respond by releasing stored insulin and continuing to make more insulin. Some evidence suggests that high SUA levels can lead to pancreatic *β*-cells damage. The damage of *β*-cells may be due to the following mechanisms: oxidative stress and inflammation induced by uric acid in *β*-cells, and inducible nitric oxide synthase gene expression induced by uric acid stimulation, resulting in nitric oxide-induced *β*-cell dysfunction [9]. SUA levels were reported to be positively correlated with *β*-cells function in diabetic subjects with normal SUA concentrations [10]. However, some cross-sectional studies have shown that SUA is independently associated with insulin resistance, but not with *β*-cells dysfunction [11, 12]. A recent study on gestational diabetes consistently showed no correlation between SUA concentrations and *β*-cells function after a 2-year follow-up of 299 female adults 1 year postpartum [13]. Thus, the relationship between HUA and *β*-cells function has not been elucidated.

The homeostasis model assessment-%B (HOMA-%B) function index is commonly used to assess islet *β*-cells function in large population cohorts, which is cheaper and simpler than the gold standard the hyperinsulinaemia euglycemic clamp technique [14]. A study showed that pancreatic *β*-cells function assessed by HOMA-%B correlated well with the results of intravenous glucose tolerance tests and has been widely used in large-scale studies [15]. However, without insulin sensitivity measurements, *β*-cells function may be misreported, as individuals with low homeostasis model assessment version 2-%B (HOMA2-%B) may be due to high sensitivity rather than *β*-cells failure [16]. On this basis, the disposition index (DI), which combines insulin sensitivity and islet secretion function, can be used to evaluate *β*-cells under compensation because it corrects insulin resistance [17]. In cohort studies, DI has been shown to decrease prior to diabetes [18].

Therefore, this cross-sectional study used the National Health and Nutrition Examination Survey (NAHENS) -nationally representative sample survey data from the United States to comprehensively evaluate the relationship between multiple indicators of pancreatic *β*-cells function and HUA.

## Materials and methods

### Study participants

The NHANES is a nationally representative health survey in the United States designed and administered by the National Center for Health Statistics (NCHS) at the Centers for Disease Control and Prevention. The NHANES uses a complex, multistage probability sampling design to represent the U.S. national, civilian and noninstitutionalized population [19]. The NCHS ethics review board has approved the NHANES protocol. Written informed consent was obtained from each participant. The institutional review board of the Guangdong Pharmaceutical University determined this study to be exempt because the data were deidentified.

**Fig. 1:**
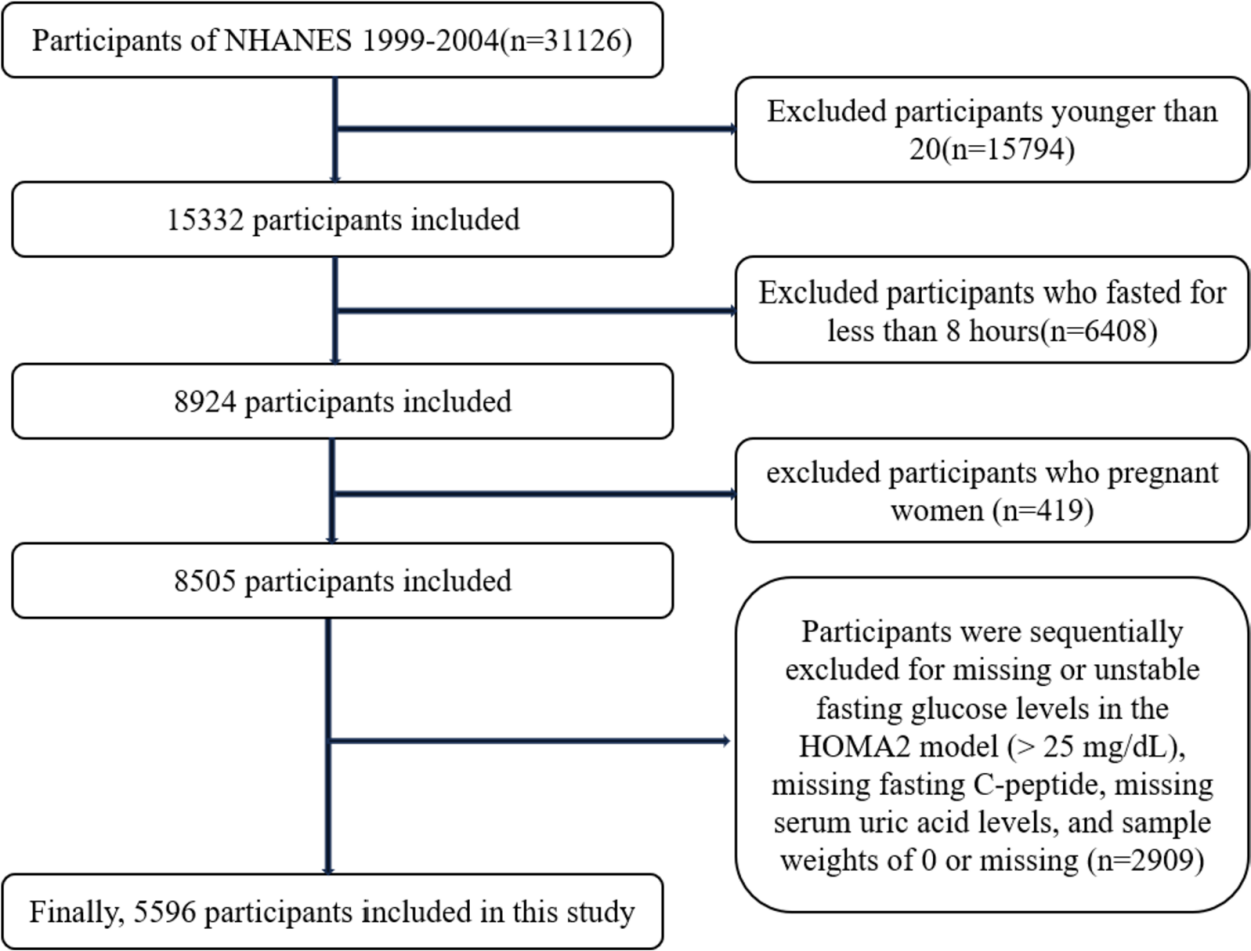
Flow chart of the screening process for the selection of eligible participants

In this cross-sectional investigation, we enrolled individuals aged 20 years and above (*n* = 15,332) who were surveyed by NHANES between 1999 and 2004. After excluding participants who had fasted for less than 8 h (*n* = 6408), as well as pregnant women (*n* = 419), participants with missing or unstable blood glucose levels in the HOMA2 model (fasting glucose levels > 25 mg/dL), subjects with missing serum C-peptide and SUA data, and participants with a sample weight of 0 or missing (*n* = 2909), a total of 5596 individuals were included in the analysis (Fig. 1).

### Ascertainment of HUA

HUA was defined as a SUA level > 7.0 mg/dL in men and > 5.7 mg/dL in women [4].

### Pancreatic *β*-cells function assessment

The C-peptide and fasting glucose data were obtained from samples of people who fasted for at least 8 h but not more than 24 h. The C-peptide and fasting glucose data from fasting samples were used to calculate HOMA2-%B and homeostasis model assessment version 2-%S (HOMA2-%S) by the homeostasis model assessment version 2 (HOMA2) calculator. HOMA2-%B was used to estimate insulin secretion and HOMA2-%S was used to evaluate insulin sensitivity. It is known that *β*-cells function can be compensatorily enhanced by environmental insulin resistance [16]. DI is an index to assess *β*-cells secretory capacity by correcting insulin resistance [15]. DI is calculated as HOMA2-%S * HOMA2-%B [15].

### Assessment of covariates

#### Race/ethnicity, family income status, education status, smoking, alcohol intake, physical activity, hypertension, diabetes, and hyperlipidemia

Information about participant race/ethnicity, family income status, education status, smoking, alcohol intake, and physical activity was collected using questionnaires. Race/ethnicity was categorized as non-Hispanic white (reference), non-Hispanic black, and other race/ethnicity. Based on SNAP eligibility, the family income status was the family income-to-poverty ratio, which can be categorized as 0.00–1.30 (reference), > 1.30–3.50, and > 3.50 and above. A higher income-to-poverty ratio indicated a higher family income status. Self-reported education status was grouped as lower than high school (reference), high school, and college or higher. In accordance with the NCHS classifications, individuals who smoked less than 100 cigarettes in their lifetime were defined as never smokers (reference); those who had smoked more than 100 cigarettes but did not smoke at the time of the survey were defined as former smokers; and those who had smoked 100 cigarettes in their lifetime and smoked cigarettes at the time of the survey were defined as current smokers. Alcohol intake was categorized as none (less than 12 alcohol drinks/lifetime) (reference), moderate (0 to 2 drinks per day for men and 0 to 1 drink per day for women), and heavy (≥ 2 drinks per day for men and ≥ 1 drinks per day for women). Physical activity was classified as active, defined as report of any regular moderate or vigorous physical activity, or inactive (reference), defined as report of no regular moderate or vigorous physical activity. Hypertension was defined as an average systolic blood pressure (SBP) ≥ 140 mmHg, an average diastolic blood pressure (DBP) ≥ 90 mmHg, or the current use of blood pressure-lowering medication [20]. Diabetes was defined as a self-reported diagnosis or treatment with hypoglycemic medications or fasting plasma glucose level ≥ 7.0 mmol/L or the Hemoglobin A1c (HbA1c) level ≥ 6.5% [21]. Hyperlipidemia is defined as total cholesterol (TC) of 200 mg/dL, triglycerides (TG) of 150 mg/dl, high-density lipoprotein (HDL) of 40 mg/dl in men, women of 50 mg/dl, or low-density lipoprotein (LDL) of 130 mg/dl. In addition, people who reported using cholesterol-lowering drugs were and those who had been told they had hyperlipidemia classified as hyperlipidemia [22].

#### TC, TG, HDL, LDL, HbA1c and estimated glomerular filtration rate

The HDL was analyzed in 1999–2002 using two methods—heparin manganese precipitation and a direct HDL immunoassay. Starting in 2003, all HDL samples were analyzed using the direct HDL immunoassay method. Triglycerides (TG) and LDL were measured by the Johns Hopkins University Lipoprotein Analytical Lab. From 1999 to 2004, HbA1c measurements were performed by the Diabetes Diagnostic Laboratory at the University of Missouri-Columbia using Primus CLC330 and Primus CLC 385. Urinary creatinine concentrations were determined using an automated colorimetric method based on a modified Jaffe reaction. The estimated glomerular filtration rate (eGFR, mL/minute/1.73 m^2^) was calculated using the Chronic Kidney Disease Epidemiology Collaboration [23].

#### Blood pressure, body mass index and waist circumference

All measurements, including blood pressure, body mass index (BMI), and waist circumference (WC), were collected during the physical examination in mobile examination centers, according to standard NHANES protocol [24], and BMI was calculated (weight in kilograms divided by height in meters squared). Blood pressure estimates were calculated by averaging three blood pressure readings.

### Statistical analysis

Following NHANES analysis guidelines [25], we incorporated combined sample weights from 1999–2004 in the statistical analysis, to account for the probability of inequality of selection, oversampling of certain sub-totals, and nonresponse adjustment. Weighted T-test was performed for continuous variables, and Rao-Scott Chi-square test was performed for categorical variables to compare means and proportions of baseline characteristics. Weighted multivariate logistic regression models were used to explore the independent association between pancreatic *β*-cells function and HUA after adjusting for potential confounders. We used weighted restricted cubic spline models fitted for logistic proportional hazards models with 4 knots at the 5th, 35th, 65th and 95th percentiles of HOMA2-%B, HOMA2-%S and DI. The reference values (O*R* = l) were set at median. Stratified analyses were also performed to examine whether this association differed by sex, age, obesity, and glycemic status. For all analyses, missing values of BMI were 118(5596), WC were 153(5596), family income status were 457(5596) and alcohol intake were 285(5596). The percentages of missing values were lower than 20%. We imputed missing data of the covariates by using multiple imputations (fully conditional specification). Five datasets were created and analyzed for sensitivity. Statistical analyses were performed using SAS, version 9.3 (SAS Institute, Inc.). The drawing was performed using the “rms” and “survey” package in R software 4.1.1. All tests were two-sided, and *P* < 0.05 was considered statistically significant.

## Results

### Basic characteristics of study participants

A total of 5596 participants were included in our analysis. 50.9% were female, 72.5% were non-Hispanic white, 53.8% had a college or higher education, 49.6% were never smokers, 62.5% were moderate drinkers, 63.4% were active, and the weighted mean age was 46.2 years (standard error (SE), 0.4). 2382, 681 and 4307 participants (weighted proportions. 35.7%, 9.8% and 75.5%) had hypertension, diabetes and hyperlipidemia, respectively. The weighted mean (SE) values of HOMA2-%B, HOMA2-%S and DI for HUA subjects were 134.9 (SE, 1.6), 50.1(SE, 1.1) and 61.8 (SE, 1.0), respectively. Patients with HUA had significantly higher HOMA2-%B and significantly lower HOMA2-%S and DI than patients without HUA (both *P* < 0.001) (Table 1). All variables showed significant differences (all *P* < 0.05) between HUA patients and patients without HUA, except race and family income status, which was older, had higher BMI, higher blood pressure, higher WC, and higher HbA1c in HUA compared to non-HUA subjects (Table 1).

**Table 1:** Demographic characteristics of U.S. adults: classified by hyperuricemia (1999–2004)

Uric Acid Status							
Characteristics^a^	Total (*n* = 5596)		Non-HUA (*n* = 4396)		HUA^b^(*n* = 1200)		*P* value
	No. (%)/Mean	SE	No. (%)/Mean	SE	No. (%)/Mean	SE	
Age, year	46.2	0.4	45.2	0.5	50.1	0.6	< 0.001
Sex							0.007
Male	2820(49.1)	0.5	2206(48.0)	0.6	614(53.2)	1.6	
Female	2776(50.9)	0.5	2190(52.0)	0.6	586(46.8)	1.6	
Race/ethnicity							0.056
Non-Hispanic white	2902(72.5)	1.9	2234(71.9)	1.8	668(74.7)	2.5	
Non-Hispanic black	995(10.5)	1.1	759(10.4)	1.1	236(10.9)	1.4	
Others	1699(17.0)	1.7	1403(17.7)	1.7	296(14.4)	2.2	
Education status							0.001
Less than high school	1779(20.2)	0.8	1395(20.1)	0.9	384(20.4)	1.3	
High school	1297(26.0)	1.0	988(24.8)	0.9	309(30.6)	1.9	
College or higher	2520(53.8)	1.2	2013(55.1)	1.3	507(49.0)	1.9	
Smoking							< 0.001
Never smoked	2820(49.6)	1.2	2248(50.4)	1.2	572(46.7)	2.0	
Formerly smoked	1545(26.1)	1.0	1135(24.3)	1.0	410(33.0)	1.8	
Currently smoked	1231(24.3)	1.0	1013(25.3)	1.2	218(20.3)	1.5	
BMI, kg/m^2^	28.1	0.1	27.1	0.1	31.6	0.3	< 0 .001
WC, cm	96.3	0.3	93.8	0.3	105.9	0.6	< 0.001
eGFR, mL/min/1.73 m^2^	98.3	0.7	100.5	0.7	89.9	1.0	< 0 .001
Alcohol intake							0.002
None	1629(27.3)	1.6	1278(26.9)	1.5	351(28.7)	2.4	
Moderate	3203(62.5)	1.4	2525(63.7)	1.4	678(58.0)	2.1	
Heavy	479(10.2)	0.7	354(9.4)	0.7	125(13.3)	1.5	
Physical activity							< 0 .001
Inactive	2451(36.6)	1.1	1875(35.3)	1.2	576(41.5)	1.6	
Active	3145(63.4)	1.1	2521(64.7)	1.2	624(58.5)	1.6	
Family income status							0.716
0.0–1.3	1377(20.2)	1.2	1075(20.3)	1.4	302(19.7)	1.4	
> 1.3–3.5	2032(37.0)	1.2	1596(36.6)	1.2	437(38.6)	2.6	
> 3.5	1730(42.8)	1.6	1360(43.1)	1.7	370(41.7)	2.6	
SBP, mmHg	122.6	0.4	121.2	0.4	128.0	0.6	< 0 .001
DBP, mmHg	72.2	0.3	71.7	0.3	74.1	0.5	< 0.001
TG, mg/dL	147.1	2.8	136.1	2.8	188.5	6.8	< 0.001
TC, mg/dL	201.4	1.0	199.4	1.0	207.9	1.7	< 0 .001
HDL, mg/dL	51.8	0.3	52.8	0.4	48.0	0.6	< 0 .001
LDL, mg/dL	120.8	0.7	119.8	0.8	125.1	1.4	0.002
Fasting blood glucose, mmol/L	5.6	0.0	5.6	0.0	5.8	0.0	< 0 .001
C- peptide, nmol/L	0.8	0.0	0.7	0.0	1.1	0.0	< 0 .001
HbA1c (%)	5.5	0.0	5.4	0.0	5.6	0.0	< 0 .001
Hypertension							< 0.001
No	3214(64.3)	1.0	2726(68.9)	1.0	488(46.8)	2.2	
Yes	2382(35.7)	1.0	1670(31.1)	1.0	712(53.2)	2.2	
Diabetes							< 0 .001
No	4915(90.2)	0.5	3926(91.7)	0.5	989(84.6)	1.3	
Yes	681(9.8)	0.5	470(8.3)	0.5	211(15.4)	1.3	
Hyperlipidemia							< 0 .001
No	1289(24.5)	0.8	1129(27.7)	1.0	160(12.6)	1.3	
Yes	4307(75.5)	0.8	3267(72.3)	1.0	1040(87.4)	1.3	
HOMA2-%B	118.3	1.0	113.9	1.0	134.9	1.6	< 0 .001
HOMA2-%S	69.9	1.1	75.2	1.4	50.1	1.1	< 0 .001
Disposition index	73.9	0.7	77.1	0.8	61.8	1.0	< 0 .001

^a^All means and SEs for continuous variables and percentages and SEs for categorical variables were weighted, with the exception of the number of participants. Because of missing values, not all totals are equal to 5,596, 4,396 and 1,200

^b^Our definition of HUA was a SUA level > 7.0 mg/dL among men and a SUA level > 5.7 mg/dL among women

### Associations between HOMA2-%B and HUA

**Fig. 2:**
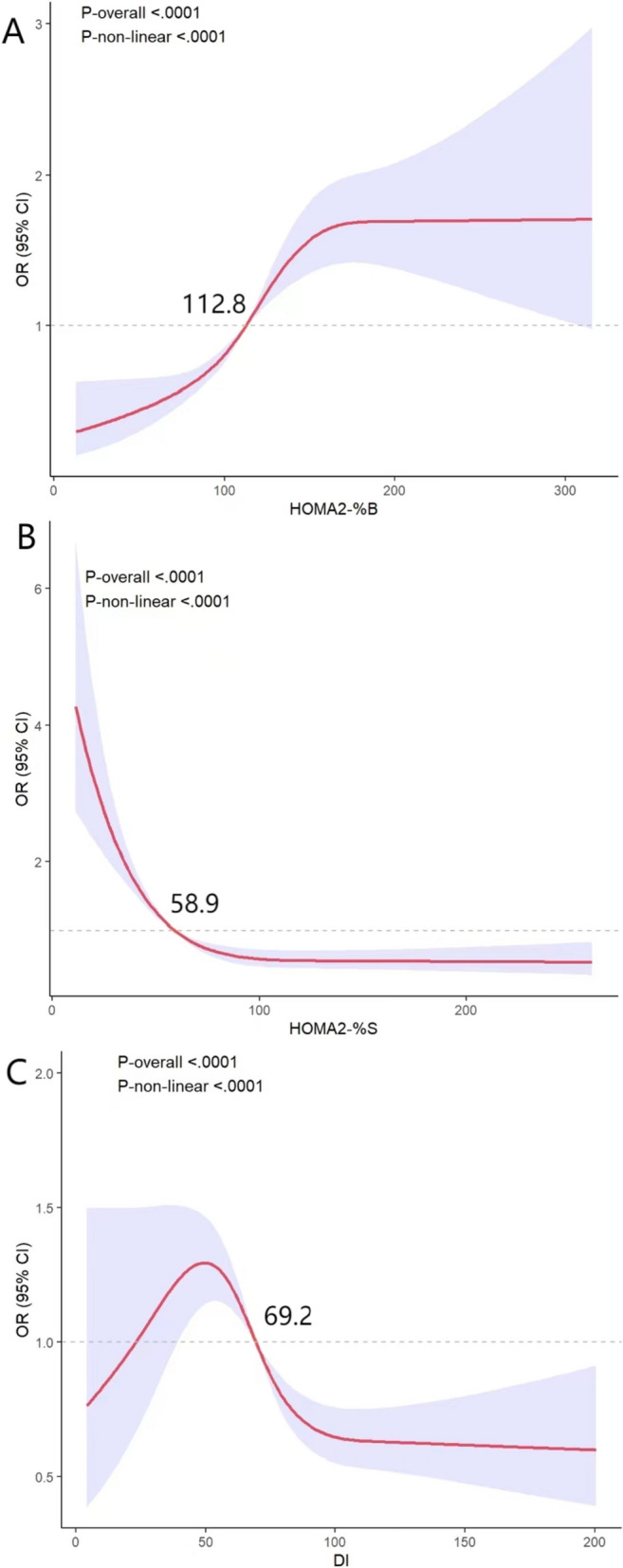
Nonlinear correlation between HOMA2-%B (**A**), HOMA2-%S (**B**), DI (**C**) and HUA for the 1999–2004 U.S. adults. The red solid lines and blue shaded areas represent the odds ratios and 95% CIs, respectively. The dotted line is O*R* = 1. The reference values (O*R* = l) were set at median. In logistic regression based on weighted restricted cubic splines, there was evidence that HOMA2-%B, HOMA2-%S and DI had nonlinear associations with HUA (all P for nonlinearity < 0.001). Models were adjusted for Age, Sex, Race/ethnicity, Education status and Family income status, Smoking, Alcohol intake, Physical activity, WC, BMI (Categorical variable: < 25, 25–30, ≥ 30), eGFR, Hypertension, Diabetes and Hyperlipidemia

Participants in the highest quartile of HOMA2-%B levels had higher odds of developing HUA (OR, 4.41; 95% CI: 3.38, 5.75) compared to subjects in the lowest quartile, and this association remained significant after adjusting for potential confounding variables (OR, 2.33; 95% CI: 1.77, 3.07 and OR, 2.55; 95% CI: 1.89, 3.43) (Table 2). SUA was positively correlated with HOMA2-%B (adjusted *r* = 0.170, *P* < 0.001) (Table S1). Regressions based on restricted cubic splines did find evidence that the relationships between HOMA2-%B and HUA were nonlinear and showed an S-shaped curve, with higher HOMA-%B increasing the risk of HUA and then levelling off, with an increased risk of HUA prevalence at outweigh 112.4 (*P* for nonlinear < 0.001) (Fig. 2A). Subgroup analyses indicated that the observed associations of HOMA2-%B with prevalence of HUA were stronger among individuals with diabetes and prediabetes compared with individuals normal (OR, 3.56 [95% CI, 1.71–7.41] and 1.86 [95% CI, 1.15–3.01] vs 1.20 [95% CI, 0.72–1.99]; *P* for interactio*n* = 0.003). The association did not significantly differ by sex, age, or BMI (Table 3).

**Table 2:** Adjusted odds ratios (95% confidence intervals) of HUA according to HOMA2-%B quartiles using weighted logistic regression

HOMA2-%B, OR (95% CI)					*P* for trend ^a^
	Q1 < 90.85	Q2 90.85–112.30	Q3 112.30–138.30	Q4 ≥ 138.30	
Model 1^b^	1.00	1.28(0.94,1.74)	1.96(1.50,2.58)	4.41(3.38,5.75)	< 0 .001
Model 2^c^	1.00	1.11(0.81,1.51)	1.40(1.05,1.87)	2.33(1.77,3.07)	< 0 .001
Model 3^d^	1.00	1.22(0.88,1.67)	1.55(1.15,2.08)	2.55(1.89,3.43)	< 0 .001

^a^Test for trend based on variable containing median value for each quartile

^b^Model 1 was adjusted for Age, Sex, Race/ethnicity, Education status and Family income status

^c^Model 2 included model 1 Smoking, Alcohol intake, Physical activity, WC, BMI (Categorical variable: < 25, 25–30, ≥ 30), eGFR

^d^Model 3 included model 2 variables plus Hypertension, Diabetes and Hyperlipidemia

**Table 3:** Adjusted odds ratios (95% confidence intervals) of HUA according to HOMA2-%B quartiles in various subpopulations^a^

HOMA2-%B, OR (95% CI)					*P* for trend ^b^	*P* for interaction
Variable	Q1	Q2	Q3	Q4		
Sex						0.559
Male	1.00	1.27(0.85–1.89)	1.68(1.17–2.41)	2.68(1.81–3.96)	< 0.001	
Female	1.00	1.17(0.71–1.94)	1.53(0.94–2.50)	2.64(1.56–4.46)	< 0.001	
Age groups, years						0.233
≤ 60	1.00	1.21(0.81–1.81)	1.38(0.97–1.97)	2.47(1.75–3.49)	< 0.001	
> 60	1.00	1.28(0.90–1.82)	2.56(1.69–3.86)	3.77(2.45–5.80)	< 0.001	
BMI (kg/m^2^)						0.918
< 25	1.00	1.27(0.71–2.28)	1.36(0.74–2.53)	2.46(1.20–5.05)	0.022	
≥ 25	1.00	1.24(0.89–1.73)	1.63(1.27–2.10)	2.66(1.94–3.64)	< 0.001	
Glycemic status						0.003
Normal	1.00	1.12(0.70–1.80)	1.20(0.72–1.99)	2.44(1.47–4.05)	< 0.001	
Prediabetes ^c^	1.00	1.18(0.65–2.14)	1.86(1.15–3.01)	3.23(1.91–5.46)	< 0.001	
Diabetes	1.00	1.81(1.03–3.19)	3.56(1.71–7.41)	1.58(0.68–3.67)	0.018	

^a^Analyses were adjusted for Age, Sex, Race/ethnicity, Education status, Family income status, Smoking, Alcohol intake, Physical activity, WC, BMI (Categorical variable: < 25, 25–30, ≥ 30), eGFR, Hypertension, Diabetes and Hyperlipidemia. Weighted logistic regression was used for analysis

^b^Test for trend based on variable containing median value for each quartile

^c^Prediabetes based on the American Diabetes Association (ADA) was defined as fasting blood glucose 5.6–6.9 mmol/L or HbA1c 5.7–6.4%

### Associations between HOMA2-%S and HUA

SUA was negatively correlated with HOMA2-%S (adjusted *r* = -0.199,* P* < 0.001) (Table S1), and the risk of the lowest quartile is higher than that of the highest quartile (OR, 8.69; 95%CI: 6.39, 11.84). In the fully adjusted model, this association is still significant (OR, 3.87; 95%CI: 2.74, 5.45) (Table 4). Based on restricted cubic spline, we found that HOMA2-%S was associated with the risk of HUA in an L-shaped curve, with higher levels of HOMA2-%S associated with a lower risk of HUA, and greater than 58.9 being a protective factor for the risk of HUA (*P* for nonlinear < 0.001) (Fig. 2B). In subgroup analyses, HOAM2-%S was found to have a significantly higher prevalence of HUA in females than in males (OR, 5.61 [95% CI, 3.15–9.96] vs 3.04 [95% CI, 2.04–4.54]; *P* for interaction < 0.001). The correlations were consistent across subgroups of different age, BMI and glycemic status (Table 5).

**Table 4:** Adjusted odds ratios (95% confidence intervals) of HUA according to HOMA2-%S quartiles using weighted logistic regression

HOMA2-%S, OR (95% CI)					*P* for trend^a^
	Q1 < 41.5	Q2 41.5–58.8	Q3 58.8–81.4	Q4 ≥ 81.4	
Model 1^b^	8.69(6.39, 11.84)	3.49(2.54, 4.81)	2.02(1.50, 2.73)	1.00	< 0 .001
Model 2^c^	4.21(3.00, 5.90)	2.08(1.50, 2.89)	1.57(1.15, 2.15)	1.00	< 0 .001
Model 3^d^	3.87(2.74, 5.45)	1.89(1.35, 2.63)	1.51(1.10, 2.07)	1.00	< 0 .001

^a^Test for trend based on variable containing median value for each quartile

^b^Model 1 was adjusted for Age, Sex, Race/ethnicity, Education status and Family income status

^c^Model 2 included model 1 Smoking, Alcohol intake, Physical activity, WC, BMI (Categorical variable: < 25, 25–30, ≥ 30), eGFR

^d^Model 3 included model 2 variables plus Hypertension, Diabetes and Hyperlipidemia

**Table 5:** Adjusted odds ratios (95% confidence intervals) of HUA according to HOMA2-%S quartiles in various subpopulations^a^

HOMA2-%S, OR (95% CI)					*P* for trend ^b^	*P* for interaction
Variable	Q1	Q2	Q3	Q4		
Sex						< 0.001
Male	3.04(2.04–4.54)	1.61(1.06–2.45)	1.36(0.93–1.99)	1.00	< 0.001	
Female	5.61(3.15–9.96)	2.29(1.36–3.87)	1.63(1.01–2.64)	1.00	< 0.001	
Age groups, years						0.372
≤ 60	3.64(2.48–5.33)	1.59(1.11–2.26)	1.33(0.94–1.87)	1.00	< 0.001	
> 60	4.96(2.76–8.92)	2.93(1.63–5.25)	2.21(1.21–4.02)	1.00	< 0.001	
BMI (kg/m^2^)						0.811
< 25	4.03(1.85–8.77)	1.62(0.81–3.25)	1.45(0.86–2.44)	1.00	0.009	
≥ 25	3.84(2.64–5.59)	1.91(1.31–2.80)	1.50(1.05–2.12)	1.00	< 0.001	
Glycemic status						0.539
Normal	4.00(2.64–6.05)	1.70(1.15–2.51)	1.42(1.02–1.97)	1.00	< 0.001	
Prediabetes ^c^	4.96(2.45–10.03)	2.30(1.13–4.67)	1.97(1.00–3.86)	1.00	< 0.001	
Diabetes	2.60(0.75–9.00)	1.61(0.41–6.30)	0.66(0.18–2.50)	1.00	0.006	

^a^Analyses were adjusted for Age, Sex, Race/ethnicity, Education status, Family income status, Smoking, Alcohol intake, Physical activity, WC, BMI (Categorical variable: < 25, 25–30, ≥ 30), eGFR, Hypertension, Diabetes and Hyperlipidemia. Weighted logistic regression was used for analysis

^b^Test for trend based on variable containing median value for each quartile

^c^Prediabetes based on the American Diabetes Association (ADA) was defined as fasting blood glucose 5.6–6.9 mmol/L or HbA1c 5.7–6.4%

### Associations between DI and HUA

Similarly, SUA was negatively correlated with DI (adjusted *r* = -0.105, *P* < 0.001) (Table S1). After fully adjusting for confounding factors, the lowest quartile of DI had a higher risk of HUA than the highest quartile (OR, 1.98; 95% CI: 1.32, 2.97) (Table 6). Evidence of a non-linear association was discovered between DI and the risk of HUA in the restricted triple sample. The highest risk of HUA prevalence was observed at a DI value of around 50, which subsequently decreased. Additionally, HUA became a protective factor at a DI value greater than 69.2 (*P* for nonlinear < 0.001) (Fig. 2C). Subgroup analyses showed that the association between DI and prevalence of HUA was higher in women than in men (OR, 2.45 [95% CI, 1.39–4.34] vs 1.58 [95% CI, 0.94–2.68]; *P* for interaction < 0.001). The correlation was not significantly different for age, BMI and glycemic status (Table 7).

**Table 6:** Adjusted odds ratios (95% confidence intervals) of HUA according to disposition index quartiles using weighted logistic regression

Disposition Index, OR (95% CI)					*P* for trend ^a^
	Q1 < 53.30	Q2 53.30–69.22	Q3 69.22–86.00	Q4 ≥ 86.00	
Model 1^b^	4.74(3.44, 6.54)	2.79(2.07, 3.76)	1.81(1.35, 2.43)	1.00	< 0 .001
Model 2^c^	2.00(1.37, 2.91)	1.49(1.09, 2.04)	1.23(0.87, 1.69)	1.00	< 0 .001
Model 3^d^	1.98(1.32, 2.97)	1.37(0.99, 1.88)	1.20(0.87, 1.65)	1.00	0.001

^a^Test for trend based on variable containing median value for each quartile

^b^Model 1 was adjusted for Age, Sex, Race/ethnicity, Education status and Family income status

^c^Model 2 included model 1 Smoking, Alcohol intake, Physical activity, WC, BMI (Categorical variable: < 25, 25–30, ≥ 30), eGFR

^d^Model 3 included model 2 variables plus Hypertension, Diabetes and Hyperlipidemia

**Table 7:** Adjusted odds ratios (95% confidence intervals) of HUA according to DI quartiles in various subpopulations^a^

Disposition Index, OR (95% CI)					*P* for trend ^b^	*P* for interaction
Variable	Q1	Q2	Q3	Q4		
Sex						< 0.001
Male	1.58(0.94–2.68)	1.07(0.65–1.76)	1.30(0.79–2.12)	1.00	0.165	
Female	2.45(1.39–4.34)	1.65(1.03–2.62)	0.91(0.61–1.37)	1.00	< 0.001	
Age groups, years						0.944
≤ 60	1.66(1.05–2.62)	1.09(0.77–1.56)	1.07(0.76–1.50)	1.00	0.064	
> 60	1.88(1.11–3.17)	1.54(0.87–2.74)	1.12(0.63–2.01)	1.00	0.005	
BMI (kg/m^2^)						0.703
< 25	1.59(0.88–2.87)	0.95(0.53–1.70)	1.28(0.72–2.27)	1.00	0.381	
≥ 25	2.08(1.31–3.29)	1.42(0.98–2.07)	1.15(0.77–1.74)	1.00	< 0.001	
Glycemic status						0.153
Normal	1.63(0.37–7.14)	1.51(1.04–2.19)	1.15(0.80–1.65)	1.00	0.049	
Prediabetes ^c^	0.85(0.40–1.79)	0.48(0.23–1.01)	0.41(0.16–1.10)	1.00	0.015	
Diabetes	2.25(0.28–18.34)	0.91(0.08–10.89)	2.53(0.23–27.89)	1.00	0.322	

^a^Analyses were adjusted for Age, Sex, Race/ethnicity, Education status, Family income status, Smoking, Alcohol intake, Physical activity, WC, BMI (Categorical variable: < 25, 25–30, ≥ 30), eGFR, Hypertension, Diabetes and Hyperlipidemia. Weighted logistic regression was used for analysis

^b^Test for trend based on variable containing median value for each quartile

^c^Prediabetes based on the American Diabetes Association (ADA) was defined as fasting blood glucose 5.6–6.9 mmol/L or HbA1c 5.7–6.4%

### Sensitivity analyses

In addition, the missing values of BMI were 118(5596), WC were 153(5596), family income status were 457(5596) and alcohol intake were 285(5596). We imputed missing data of the covariates by using multiple imputations. Five datasets were created and analyzed together. Overall, the results of our sensitivity analyses were consistent with those of our primary analysis (Tables S2-6 for HOMA2-%B, Tables S7-11 for HOMA2-%S, Tables S12-16 for DI in the Supplementary Material).

## Discussion

This is the first study exploring the nonlinear correlation between pancreatic *β*-cells function and HUA, based on the NHANES. Our study revealed that HOMA2-%B, HOMA2-%S and DI were all closely associated with HUA. This relationship was independent of age, sex, race/ethnicity, education status, family income status, smoking, alcohol intake, physical activity, WC, BMI, eGFR, hypertension, diabetes and hyperlipidemia. Furthermore, we found that the associations of HOMA2-%B with prevalence of HUA were stronger among individuals with diabetes and prediabetes compared with individuals normal, and the association of HOMA2-%S and DI with the prevalence of HUA was stronger in women than in men.

The level of pancreatic *β*-cells function is an important indicator of health and is essential to avoid type 2 diabetes, hypertension and cardiovascular disease. In this study, the weighted mean of HOMA2-%B was 118.1 in U.S. adults, higher than those reported in Korea (100.09) [26] and China (84.34) [27]. The differences between countries may be due to ethnic differences, as well as the level of health care in each country. Furthermore, in an analysis of NHANES 2001–2016 trends in *β*-cells dysfunction, there was a trend toward increased HOMA2-%B in both sexes in the pre-diabetes group [28]. This suggests that *β*-cells dysfunction is a potential public health hazard of concern in the United States. In this study, participants with higher HOMA2-%B were more likely to develop HUA, but after correction of insulin resistance, participants with lower DI were more likely to develop HUA. Our findings are consistent with trials reporting pancreatic *β*-cells death and dysfunction due to HUA [29]. A study of type 2 diabetes consistently showed that after an oral glucose tolerance test in 1,021 patients with type 2 diabetes, patients with higher SUA had greater insulin-secreting capacity early in the disease than those with lower SUA, but their residual *β*-cells function deteriorated at an accelerated rate [30]. Although many epidemiological studies have explored the relationship between *β*-cells function and HUA, previous studies have focused more on the impact of elevated SUA levels on insulin resistance. Our study adds another perspective that insulin function indicators can be used as independent predictors of HUA. Improving insulin resistance will provide new possibilities for controlling and delaying the occurrence and development of SUA.

However, the evidence that support the possibility of an increased prevalence of HUA associated with *β*-cells dysfunction is limited and controversial. A cross-sectional study of the China Health and Nutrition Examination Survey exhibited no significant association between SUA and *β*-cells dysfunction in non-diabetic patients [12]. The similar result is found in another cross-sectional study of adults at risk for type 2 diabetes [11]. The apparent controversy in these studies stems from several factors. For one thing, the populations in these studies were ethnically diverse, which may have contributed to the discrepancy. In addition, these studies all used insulin to calculate *β*-cells function, but insulin circulates through the liver and is susceptible to exogenous insulin. Third, the measures of pancreatic *β*-cells function calculated in these studies did not take into account the effect of insulin resistance, which could lead to bias. Here, we use C-peptide, which is more stable than insulin, to accurately assess *β*-cells function, and use DI, a marker of insulin resistance corrected, to explore the relationship between HUA and *β*-cells function. However, in the case of insulin resistance, the enhanced *β*-cells response did not eliminate the effects of insulin resistance, and we found that DI was negatively correlated with the presence of HUA. With the exception of the one animal study [29], all population studies mentioned above were cross-sectional or case–control studies, with lack of evidence of causal correlation between *β*-cells function and HUA. More large-scale prospective studies are needed to confirm our findings.

We found a non-linear association between pancreatic *β*-cells function and HUA. Insulin sensitivity and secretion are joined by a negative feedback loop characterized by a hyperbolic function. Shifts in insulin sensitivity or resistance are accompanied by compensatory alterations in *β*-cells function [31]. The curve results of this study visually display an opposite association with HUA risk between insulin sensitivity and insulin secretion, reflecting the interdependent and interactive relationship between them in the ability to handle blood glucose. As far as we know, this is the first study to illustrate the non-linear relationship between *β*-cells function and HUA. Furthermore, our study adds a new insight that DI, as a more comprehensive *β*-cells function indicator in the context of insulin resistance, could be a more accurate predictor for the presence of HUA than the application of a single indicator evaluation (either HOMA2-%B or HOMA2-%S). When insulin resistance occurs, insulin function becomes hyperactive, but as long as the DI index remains within the appropriate range, the risk of HUA might not increase. On the contrary, if the increase of insulin secretion cannot fully compensate for insulin resistance, DI index will decline, and abnormal SUA metabolism and HUA might occur.

Although the mechanisms underlying the link between *β*-cells dysfunction and HUA are unclear, evidence from physiological experiments may provide some clues. First, insulin resistance is common in pre-islet *β*-cells dysfunction. In insulin-resistant patients, elevated serum insulin leads to increased SUA reabsorption by proximal renal tubules, resulting in reduced SUA excretion and raised SUA levels [32]. A bidirectional Mendelian randomization trial reported that hyperinsulinemia leads to HUA, not the other way around [33]. Second, SUA and glucose share a common transporter protein (GLUT9) [34, 35], hyperglycemia may up-regulate the level of GLUT9 protein in the kidney and increase the reabsorption of SUA in the kidney [36].

In our study, the association between HOMA2-%S, DI and the prevalence of HUA was found to be stronger in women than in men. This sex difference was found by multiple regression analysis and confirmed by a significant interaction term (sex × HOMA2-%S, sex × DI). Although the cause of these sex differences is unknown, sex hormones may play a vital role. Metabolic changes during the menopause may lead to increased SUA levels in women [37], and epidemiological studies have also shown a positive association between SUA and insulin resistance in older women, but not in men [38]. Consistent with this gender difference, many studies have found that serum uric acid levels are more strongly associated with disease in women, including chronic kidney disease [39] and coronary heart disease [37]. More research is needed into the sex-specific role of *β*-cells function in HUA. Furthermore, we found a stronger association between HOMA2-%B and HUA in diabetic and prediabetic populations than in normal populations. This phenomenon can be well explained by the predictive value of DI for the risk of hyperuricemia found in this study. Diabetic and pre-diabetic populations have reduced insulin sensitivity compared to normal populations, and even with the same HOMA2-%B levels, the latter have a worse glycemic disposition and a higher risk of developing HUA.

Our study has some major strengths. The population-based design, multistage sampling and the large sample size strengthen the representativeness of our findings in the US population. On the other hand, we calculated DI based on the HOMA2 model because Matthews et al. recommended computerized HOMA2 as the standard method for HOMA rather than the original HOMA1 [16]. A Korean study showed that HOMA2 was a better predictor of diabetes progression than HOMA1 in pre-diabetic or non-diabetic patients [26]. More importantly, DI assesses pancreatic *β*-cells function more accurately than HOMA2-%B because it corrected for insulin sensitivity levels [40]. A study showed that the predictive power of DI for type 2 diabetes was higher than that of HOMA2-%B [41]. In addition, we used fasting serum C-peptide data instead of insulin data to calculate *β*-cells function. This can be explained by the fact that the insulin concentration assessed in peripheral blood does not fully reflect the insulin secreted by the pancreas. Insulin is partially cleared in the portal vein before entering the peripheral circulation, whereas C-peptide and insulin are co-secreted in equal amounts and are not degraded by the liver [42, 43].

This study has several limitations. First, due to the cross-sectional design, the association between *β*-cells dysfunction and HUA is hardly likely to be causal. Second, as an observational study, there may be potential residual confounding factors. Third, we assessed *β*-cells function based on the HOMA2 model, which is simple to use but less accurate in reflecting *β*-cells function than other measures of the hyperglycemic clamp method (the gold standard) [44]. Finally, this study was conducted in the United States, so we cannot extend the conclusion to other ethnic groups. Therefore, our findings should be replicated in future studies in other populations and further cohort studies established to determine the causal relationship between *β*-cells function and the presence of HUA.

## Conclusions

Our study showed that HUA was positively associated with HOMA2-%B but negatively associated with HOMA2-%S and DI in U.S. adults. Altogether, these results suggest that insufficient pancreatic *β*-cells function might be a new predictor for the presence of HUA and should be validated in future large prospective studies in different populations.

### Supplementary Information

**Additional file 1:** **Table S1.** Partial Correlation Coefficients Between SUA and Indexes. **Table S2. **Adjusted odds ratios (95% confidence intervals) of HUA according to HOMA2-%B quartiles using weighted logistic regression (multiple imputations dataset 1). **Table S3. **Adjusted odds ratios (95% confidence intervals) of HUA according to HOMA2-%B quartiles using weighted logistic regression (multiple imputations dataset 2). **Table S4. **Adjusted odds ratios (95% confidence intervals) of HUA according to HOMA2-%B quartiles using weighted logistic regression (multiple imputations dataset 3). **Table S5. **Adjusted odds ratios (95% confidence intervals) of HUA according to HOMA2-%B quartiles using weighted logistic regression (multiple imputations dataset 4). **Table S6. **Adjusted odds ratios (95% confidence intervals) of HUA according to HOMA2-%B quartiles using weighted logistic regression (multiple imputations dataset 5). **Table S7. **Adjusted odds ratios (95% confidence intervals) of HUA according to HOMA2-%S quartiles using weighted logistic regression (multiple imputations dataset 1). **Table S8. **Adjusted odds ratios (95% confidence intervals) of HUA according to HOMA2-%S quartiles using weighted logistic regression (multiple imputations dataset 2). **Table S9. **Adjusted odds ratios (95% confidence intervals) of HUA according to HOMA2-%S quartiles using weighted logistic regression (multiple imputations dataset 3). **Table S10. **Adjusted odds ratios (95% confidence intervals) of HUA according to HOMA2-%S quartiles using weighted logistic regression (multiple imputations dataset 4). **Table S11. **Adjusted odds ratios (95% confidence intervals) of HUA according to HOMA2-%S quartiles using weighted logistic regression (multiple imputations dataset 5). **Table S12. **Adjusted odds ratios (95% confidence intervals) of HUA according to disposition index quartiles using weighted logistic regression (multiple imputations dataset 1). **Table S13. **Adjusted odds ratios (95% confidence intervals) of HUA according to disposition index quartiles using weighted logistic regression (multiple imputations dataset 2). **Table S14. **Adjusted odds ratios (95% confidence intervals) of HUA according to disposition index quartiles using weighted logistic regression (multiple imputations dataset 3). **Table S15. **Adjusted odds ratios (95% confidence intervals) of HUA according to disposition index quartiles using weighted logistic regression (multiple imputations dataset 4). **Table S16. **Adjusted odds ratios (95% confidence intervals) of HUA according to disposition index quartiles using weighted logistic regression (multiple imputations dataset 5).

## Data Availability

The dataset(s) supporting the conclusions of this article is(are) available in the NHANES repository, https://www.cdc.gov/nchs/nhanes/index.htm.

## Abbreviations

ADA: American Diabetes Association
BMI: Body mass index
DBP: Diastolic pressure
DI: Disposition index
eGFR: Estimated glomerular filtration rate
HbA1c: Hemoglobin A1c
HDL: High density lipoprotein
HOMA2: Homeostasis model assessment version 2
HOMA2-%B: Homeostasis model assessment version 2-%B
HOMA2-%S: Homeostasis model assessment version 2-%S
HUA: Hyperuricemia
LDL: Low density lipoprotein
NCHS: National Center for Health Statistics
NHANES: National Health and Nutrition Survey
SBP: Systolic pressure
SE: Standard error
SUA: Serum uric acid
TG: Triglyceride
TC: Total cholesterol
WC: Waist circumference
