# Supplementary material for: Insufficient compensatory pancreatic β-cells function might be closely associated with hyperuricemia in U.S. adults: evidence from the National Health and Nutrition Examination Survey
Source: BMC Public Health. 2024 Jan 3;24:85. doi: 10.1186/s12889-023-17471-0 (PMC10765924; doi:10.1186/s12889-023-17471-0)
Supplement: Supplementary file 1 — Additional file 1: Table S1. Partial Correlation Coefficients Between SUA and Indexes. Table S2. Adjusted odds ratios (95% confidence intervals) of HUA according to HOMA2-%B quartiles using weighted logistic regression (multiple imputations dataset 1). Table S3. Adjusted odds ratios (95% confidence intervals) of HUA according to HOMA2-%B quartiles using weighted logistic regression (multiple imputations dataset 2). Table S4. Adjusted odds ratios (95% confidence intervals) of HUA according to HOMA2-%B quartiles using weighted logistic regression (multiple imputations dataset 3). Table S5. Adjusted odds ratios (95% confidence intervals) of HUA according to HOMA2-%B quartiles using weighted logistic regression (multiple imputations dataset 4). Table S6. Adjusted odds ratios (95% confidence intervals) of HUA according to HOMA2-%B quartiles using weighted logistic regression (multiple imputations dataset 5). Table S7. Adjusted odds ratios (95% confidence intervals) of HUA according to HOMA2-%S quartiles using weighted logistic regression (multiple imputations dataset 1). Table S8. Adjusted odds ratios (95% confidence intervals) of HUA according to HOMA2-%S quartiles using weighted logistic regression (multiple imputations dataset 2). Table S9. Adjusted odds ratios (95% confidence intervals) of HUA according to HOMA2-%S quartiles using weighted logistic regression (multiple imputations dataset 3). Table S10. Adjusted odds ratios (95% confidence intervals) of HUA according to HOMA2-%S quartiles using weighted logistic regression (multiple imputations dataset 4). Table S11. Adjusted odds ratios (95% confidence intervals) of HUA according to HOMA2-%S quartiles using weighted logistic regression (multiple imputations dataset 5). Table S12. Adjusted odds ratios (95% confidence intervals) of HUA according to disposition index quartiles using weighted logistic regression (multiple imputations dataset 1). Table S13. Adjusted odds ratios (95% confidence intervals) of HUA according [file 12889_2023_17471_MOESM1_ESM.docx]

**Supplementary material**

**Table S1** Partial Correlation Coefficients Between SUA and Indexes

|  | Correlation Coefficient | *P* value | Adjusted Correlation Coefficient ^a^ | *P* value |
| --- | --- | --- | --- | --- |
| HOMA2-%B | 0.192 | <0.001 | 0.170 | <0.001 |
| HOMA2-%S | -0.339 | <0.001 | -0.199 | <0.001 |
| DI | -0.285 | <0.001 | -0.105 | <0.001 |

^a^ Analyses were adjusted for Age, Sex, Race/ethnicity, Education status, Family income status, smoking, Alcohol intake, Physical activity, WC, BMI (Categorical variable: <25, 25-30, ≥30), eGFR, hypertension, Diabetes and Hyperlipidemia.

**Table S2.** Adjusted odds ratios (95% confidence intervals) of HUA according to HOMA2-%B quartiles using weighted logistic regression (multiple imputations dataset 1).

| HOMA2-%B，OR (95% CI) | | | | | *P* for trend ^a^ |
| --- | --- | --- | --- | --- | --- |
|  | Q1  < 90.85 | Q2  90.85-112.30 | Q3  112.30-138.30 | Q4  ≥ 138.30 |  |
| Model 1^b^ | 1.00 | 1.31(0.97,1.77) | 1.98(1.56,2.52) | 4.28(3.33,5.52) | <0 .001 |
| Model 2^c^ | 1.00 | 1.18(0.86,1.61) | 1.42(1.09,1.86) | 2.21(1.71,2.87) | <0 .001 |
| Model 3^d^ | 1.00 | 1.28(0.92,1.76) | 1.54(1.18,2.01) | 2.36(1.80,3.10) | <0 .001 |

^a^ Test for trend based on variable containing median value for each quartile.

^b^ Model 1 was adjusted for Age, Sex, Race/ethnicity, Education status and Family income status.

^c^ Model 2 included model 1 Smoking, Alcohol intake, Physical activity, WC, BMI (Categorical variable: <25, 25-30, ≥30), eGFR.

^d^ Model 3 included model 2 variables plus Hypertension, Diabetes and Hyperlipidemia.

**Table S3.** Adjusted odds ratios (95% confidence intervals) of HUA according to HOMA2-%B quartiles using weighted logistic regression (multiple imputations dataset 2).

| HOMA2-%B，OR (95% CI) | | | | | *P* for trend ^a^ |
| --- | --- | --- | --- | --- | --- |
|  | Q1  < 90.85 | Q2  90.85-112.30 | Q3  112.30-138.30 | Q4  ≥ 138.30 |  |
| Model 1^b^ | 1.00 | 1.30(0.96,1.75) | 1.98(1.55,2.52) | 4.28(3.34,5.50) | <0 .001 |
| Model 2^c^ | 1.00 | 1.18(0.87,1.60) | 1.50(1.15,1.96) | 2.44(1.89,3.16) | <0 .001 |
| Model 3^d^ | 1.00 | 1.31(0.96,1.79) | 1.67(1.28,2.17) | 2.69(2.07,3.51) | <0 .001 |

^a^ Test for trend based on variable containing median value for each quartile.

^b^ Model 1 was adjusted for Age, Sex, Race/ethnicity, Education status and Family income status.

^c^ Model 2 included model 1 Smoking, Alcohol intake, Physical activity, WC, BMI (Categorical variable: <25, 25-30, ≥30), eGFR.

^d^ Model 3 included model 2 variables plus Hypertension, Diabetes and Hyperlipidemia.

**Table S4.** Adjusted odds ratios (95% confidence intervals) of HUA according to HOMA2-%B quartiles using weighted logistic regression (multiple imputations dataset 3).

| HOMA2-%B，OR (95% CI) | | | | | *P* for trend ^a^ |
| --- | --- | --- | --- | --- | --- |
|  | Q1  < 90.85 | Q2  90.85-112.30 | Q3  112.30-138.30 | Q4  ≥ 138.30 |  |
| Model 1^b^ | 1.00 | 1.29(0.96,1.75) | 1.98(1.55,2.53) | 4.31(3.36,5.52) | <0 .001 |
| Model 2^c^ | 1.00 | 1.19(0.87,1.62) | 1.46(1.12,1.90) | 2.30(1.79,2.97) | <0 .001 |
| Model 3^d^ | 1.00 | 1.30(0.95,1.79) | 1.59(1.23,2.07) | 2.51(1.92,3.26) | <0 .001 |

^a^ Test for trend based on variable containing median value for each quartile.

^b^ Model 1 was adjusted for Age, Sex, Race/ethnicity, Education status and Family income status.

^c^ Model 2 included model 1 Smoking, Alcohol intake, Physical activity, WC, BMI (Categorical variable: <25, 25-30, ≥30), eGFR.

^d^ Model 3 included model 2 variables plus Hypertension, Diabetes and Hyperlipidemia.

**Table S5.** Adjusted odds ratios (95% confidence intervals) of HUA according to HOMA2-%B quartiles using weighted logistic regression (multiple imputations dataset 4).

| HOMA2-%B，OR (95% CI) | | | | | *P* for trend ^a^ |
| --- | --- | --- | --- | --- | --- |
|  | Q1  < 90.85 | Q2  90.85-112.30 | Q3  112.30-138.30 | Q4  ≥ 138.30 |  |
| Model 1^b^ | 1.00 | 1.30(0.96,1.75) | 1.97(1.55,2.52) | 4.28(3.34,5.49) | <0 .001 |
| Model 2^c^ | 1.00 | 1.19(0.87,1.62) | 1.46(1.12,1.91) | 2.33(1.80,3.01) | <0 .001 |
| Model 3^d^ | 1.00 | 1.29(0.94,1.78) | 1.59(1.22,2.07) | 2.51(1.92,3.27) | <0 .001 |

^a^ Test for trend based on variable containing median value for each quartile.

^b^ Model 1 was adjusted for Age, Sex, Race/ethnicity, Education status and Family income status.

^c^ Model 2 included model 1 Smoking, Alcohol intake, Physical activity, WC, BMI (Categorical variable: <25, 25-30, ≥30), eGFR.

^d^ Model 3 included model 2 variables plus Hypertension, Diabetes and Hyperlipidemia.

**Table S6.** Adjusted odds ratios (95% confidence intervals) of HUA according to HOMA2-%B quartiles using weighted logistic regression (multiple imputations dataset 5).

| HOMA2-%B，OR (95% CI) | | | | | *P* for trend ^a^ |
| --- | --- | --- | --- | --- | --- |
|  | Q1  < 90.85 | Q2  90.85-112.30 | Q3  112.30-138.30 | Q4  ≥ 138.30 |  |
| Model 1^b^ | 1.00 | 1.33(0.98,1.79) | 2.01(1.58,2.56) | 4.35(3.36,5.63) | <0 .001 |
| Model 2^c^ | 1.00 | 1.19(0.87,1.63) | 1.44(1.10,1.89) | 2.27(1.74,2.97) | <0 .001 |
| Model 3^d^ | 1.00 | 1.29(0.93,1.78) | 1.56(1.19,2.04) | 2.43(1.84,3.21) | <0 .001 |

^a^ Test for trend based on variable containing median value for each quartile.

^b^ Model 1 was adjusted for Age, Sex, Race/ethnicity, Education status and Family income status.

^c^ Model 2 included model 1 Smoking, Alcohol intake, Physical activity, WC, BMI (Categorical variable: <25, 25-30, ≥30), eGFR.

^d^ Model 3 included model 2 variables plus Hypertension, Diabetes and Hyperlipidemia.

**Table S7.** Adjusted odds ratios (95% confidence intervals) of HUA according to HOMA2-%S quartiles using weighted logistic regression (multiple imputations dataset 1).

| HOMA2-%S，OR (95% CI) | | | | | *P* for trend ^a^ |
| --- | --- | --- | --- | --- | --- |
|  | Q1  < 41.5 | Q2  41.5-58.8 | Q3  58.8-81.4 | Q4  ≥81.4 |  |
| Model 1^b^ | 8.87(6.61, 11.90) | 3.62(2.66, 4.94) | 2.05(1.54, 2.74) | 1.00 | <0 .001 |
| Model 2^c^ | 3.62(2.68, 4.90) | 1.89(1.38, 2.60) | 1.45(1.09, 1.95) | 1.00 | <0 .001 |
| Model 3^d^ | 3.31(2.42, 4.52) | 1.72(1.24, 2.38) | 1.39(1.03, 1.86) | 1.00 | <0 .001 |

^a^ Test for trend based on variable containing median value for each quartile.

^b^ Model 1 was adjusted for Age, Sex, Race/ethnicity, Education status and Family income status.

^c^ Model 2 included model 1 Smoking, Alcohol intake, Physical activity, WC, BMI (Categorical variable: <25, 25-30, ≥30), eGFR.

^d^ Model 3 included model 2 variables plus Hypertension, Diabetes and Hyperlipidemia.

**Table S8.** Adjusted odds ratios (95% confidence intervals) of HUA according to HOMA2-%S quartiles using weighted logistic regression (multiple imputations dataset 2).

| HOMA2-%S，OR (95% CI) | | | | | *P* for trend ^a^ |
| --- | --- | --- | --- | --- | --- |
|  | Q1  < 41.5 | Q2  41.5-58.8 | Q3  58.8-81.4 | Q4  ≥81.4 |  |
| Model 1^b^ | 8.87(6.64, 11.85) | 3.62(2.66, 4.92) | 2.05(1.54, 2.74) | 1.00 | <0 .001 |
| Model 2^c^ | 4.63(3.40, 6.32) | 2.31(1.66, 3.21) | 1.63(1.21, 2.20) | 1.00 | <0 .001 |
| Model 3^d^ | 4.24(3.05, 5.90) | 2.10(1.49, 2.96) | 1.56(1.15, 2.12) | 1.00 | <0 .001 |

^a^ Test for trend based on variable containing median value for each quartile.

^b^ Model 1 was adjusted for Age, Sex, Race/ethnicity, Education status and Family income status.

^c^ Model 2 included model 1 Smoking, Alcohol intake, Physical activity, WC, BMI (Categorical variable: <25, 25-30, ≥30), eGFR.

^d^ Model 3 included model 2 variables plus Hypertension, Diabetes and Hyperlipidemia.

**Table S9.** Adjusted odds ratios (95% confidence intervals) of HUA according to HOMA2-%S quartiles using weighted logistic regression (multiple imputations dataset 3).

| HOMA2-%S，OR (95% CI) | | | | | *P* for trend ^a^ |
| --- | --- | --- | --- | --- | --- |
|  | Q1  < 41.5 | Q2  41.5-58.8 | Q3  58.8-81.4 | Q4  ≥81.4 |  |
| Model 1^b^ | 8.88(6.64, 11.87) | 3.62(2.67, 4.91) | 2.06(1.54, 2.74) | 1.00 | <0 .001 |
| Model 2^c^ | 3.96(2.94, 5.35) | 2.03(1.48, 2.79) | 1.53(1.14, 2.06) | 1.00 | <0 .001 |
| Model 3^d^ | 3.64(2.66, 4.99) | 1.85(1.33, 2.57) | 1.46(1.08, 1.98) | 1.00 | <0 .001 |

^a^ Test for trend based on variable containing median value for each quartile.

^b^ Model 1 was adjusted for Age, Sex, Race/ethnicity, Education status and Family income status.

^c^ Model 2 included model 1 Smoking, Alcohol intake, Physical activity, WC, BMI (Categorical variable: <25, 25-30, ≥30), eGFR.

^d^ Model 3 included model 2 variables plus Hypertension, Diabetes and Hyperlipidemia.

**Table S10.** Adjusted odds ratios (95% confidence intervals) of HUA according to HOMA2-%S quartiles using weighted logistic regression (multiple imputations dataset 4).

| HOMA2-%S，OR (95% CI) | | | | | *P* for trend ^a^ |
| --- | --- | --- | --- | --- | --- |
|  | Q1  < 41.5 | Q2  41.5-58.8 | Q3  58.8-81.4 | Q4  ≥81.4 |  |
| Model 1^b^ | 8.85(6.63, 11.82) | 3.61(2.66, 4.91) | 2.05(1.54, 2.74) | 1.00 | <0 .001 |
| Model 2^c^ | 3.97(2.89, 5.45) | 2.06(1.48, 2.88) | 1.54(1.13, 2.08) | 1.00 | <0 .001 |
| Model 3^d^ | 3.65(2.62, 5.10) | 1.88(1.33, 2.65) | 1.47(1.08, 2.00) | 1.00 | <0 .001 |

^a^ Test for trend based on variable containing median value for each quartile.

^b^ Model 1 was adjusted for Age, Sex, Race/ethnicity, Education status and Family income status.

^c^ Model 2 included model 1 Smoking, Alcohol intake, Physical activity, WC, BMI (Categorical variable: <25, 25-30, ≥30), eGFR.

^d^ Model 3 included model 2 variables plus Hypertension, Diabetes and Hyperlipidemia.

**Table S11.** Adjusted odds ratios (95% confidence intervals) of HUA according to HOMA2-%S quartiles using weighted logistic regression (multiple imputations dataset 5).

| HOMA2-%S，OR (95% CI) | | | | | *P* for trend ^a^ |
| --- | --- | --- | --- | --- | --- |
|  | Q1  < 41.5 | Q2  41.5-58.8 | Q3  58.8-81.4 | Q4  ≥81.4 |  |
| Model 1^b^ | 8.82(6.58, 11.83) | 3.63(2.66, 4.97) | 2.06(1.54, 2.77) | 1.00 | <0 .001 |
| Model 2^c^ | 3.67(2.69, 5.02) | 1.94(1.40, 2.68) | 1.47(1.08, 2.00) | 1.00 | <0 .001 |
| Model 3^d^ | 3.39(2.45, 4.70) | 1.77(1.26, 2.47) | 1.41(1.03, 1.92) | 1.00 | <0 .001 |

^a^ Test for trend based on variable containing median value for each quartile.

^b^ Model 1 was adjusted for Age, Sex, Race/ethnicity, Education status and Family income status.

^c^ Model 2 included model 1 Smoking, Alcohol intake, Physical activity, WC, BMI (Categorical variable: <25, 25-30, ≥30), eGFR.

^d^ Model 3 included model 2 variables plus Hypertension, Diabetes and Hyperlipidemia.

**Table S12.** Adjusted odds ratios (95% confidence intervals) of HUA according to disposition index quartiles using weighted logistic regression (multiple imputations dataset 1).

| Disposition Index，OR (95% CI) | | | | | *P* for trend ^a^ |
| --- | --- | --- | --- | --- | --- |
|  | Q1  < 53.30 | Q2  53.30-69.22 | Q3  69.22-86.00 | Q4  ≥ 86.00 |  |
| Model 1^b^ | 4.83(3.52, 6.63) | 2.87(2.15, 3.84) | 1.82(1.37, 2.43) | 1.00 | <0 .001 |
| Model 2^c^ | 1.92(1.37, 2.69) | 1.45(1.09, 1.93) | 1.22(0.90, 1.65) | 1.00 | <0 .001 |
| Model 3^d^ | 1.90(1.31, 2.75) | 1.33(0.99, 1.79) | 1.19(0.88, 1.61) | 1.00 | <0 .001 |

^a^ Test for trend based on variable containing median value for each quartile.

^b^ Model 1 was adjusted for Age, Sex, Race/ethnicity, Education status and Family income status.

^c^ Model 2 included model 1 Smoking, Alcohol intake, Physical activity, WC, BMI (Categorical variable: <25, 25-30, ≥30), eGFR.

^d^ Model 3 included model 2 variables plus Hypertension, Diabetes and Hyperlipidemia.

**Table S13.** Adjusted odds ratios (95% confidence intervals) of HUA according to disposition index quartiles using weighted logistic regression (multiple imputations dataset 2).

| Disposition Index，OR (95% CI) | | | | | *P* for trend ^a^ |
| --- | --- | --- | --- | --- | --- |
|  | Q1  < 53.30 | Q2  53.30-69.22 | Q3  69.22-86.00 | Q4  ≥ 86.00 |  |
| Model 1^b^ | 4.86(3.54, 6.67) | 2.87(2.14, 3.86) | 1.82(1.36, 2.43) | 1.00 | <0 .001 |
| Model 2^c^ | 2.14(1.50, 3.05) | 1.61(1.18, 2.18) | 1.29(0.93, 1.79) | 1.00 | <0 .001 |
| Model 3^d^ | 2.10(1.42, 3.09) | 1.47(1.08, 2.01) | 1.25(0.91, 1.73) | 1.00 | <0 .001 |

^a^ Test for trend based on variable containing median value for each quartile.

^b^ Model 1 was adjusted for Age, Sex, Race/ethnicity, Education status and Family income status.

^c^ Model 2 included model 1 Smoking, Alcohol intake, Physical activity, WC, BMI (Categorical variable: <25, 25-30, ≥30), eGFR.

^d^ Model 3 included model 2 variables plus Hypertension, Diabetes and Hyperlipidemia.

**Table S14.** Adjusted odds ratios (95% confidence intervals) of HUA according to disposition index quartiles using weighted logistic regression (multiple imputations dataset 3).

| Disposition Index，OR (95% CI) | | | | | *P* for trend ^a^ |
| --- | --- | --- | --- | --- | --- |
|  | Q1  < 53.30 | Q2  53.30-69.22 | Q3  69.22-86.00 | Q4  ≥ 86.00 |  |
| Model 1^b^ | 4.81(3.50, 6.62) | 2.87(2.14, 3.85) | 1.80(1.35, 2.42) | 1.00 | <0 .001 |
| Model 2^c^ | 1.95(1.39, 2.75) | 1.49(1.11, 2.00) | 1.24(0.91, 1.70) | 1.00 | <0 .001 |
| Model 3^d^ | 1.94(1.33, 2.84) | 1.37(1.02, 1.86) | 1.21(0.89, 1.66) | 1.00 | <0 .001 |

^a^ Test for trend based on variable containing median value for each quartile.

^b^ Model 1 was adjusted for Age, Sex, Race/ethnicity, Education status and Family income status.

^c^ Model 2 included model 1 Smoking, Alcohol intake, Physical activity, WC, BMI (Categorical variable: <25, 25-30, ≥30), eGFR.

^d^ Model 3 included model 2 variables plus Hypertension, Diabetes and Hyperlipidemia.

**Table S15.** Adjusted odds ratios (95% confidence intervals) of HUA according to disposition index quartiles using weighted logistic regression (multiple imputations dataset 4).

| Disposition Index，OR (95% CI) | | | | | *P* for trend ^a^ |
| --- | --- | --- | --- | --- | --- |
|  | Q1  < 53.30 | Q2  53.30-69.22 | Q3  69.22-86.00 | Q4  ≥ 86.00 |  |
| Model 1^b^ | 4.84(3.52, 6.65) | 2.86(2.14, 3.84) | 1.81(1.36, 2.42) | 1.00 | <0 .001 |
| Model 2^c^ | 1.97(1.39, 2.79) | 1.51(1.12, 2.04) | 1.25(0.91, 1.72) | 1.00 | <0 .001 |
| Model 3^d^ | 1.97(1.34, 2.89) | 1.39(1.02, 1.89) | 1.22(0.89, 1.68) | 1.00 | <0 .001 |

^a^ Test for trend based on variable containing median value for each quartile.

^b^ Model 1 was adjusted for Age, Sex, Race/ethnicity, Education status and Family income status.

^c^ Model 2 included model 1 Smoking, Alcohol intake, Physical activity, WC, BMI (Categorical variable: <25, 25-30, ≥30), eGFR.

^d^ Model 3 included model 2 variables plus Hypertension, Diabetes and Hyperlipidemia.

**Table S16.** Adjusted odds ratios (95% confidence intervals) of HUA according to disposition index quartiles using weighted logistic regression (multiple imputations dataset 5).

| Disposition Index，OR (95% CI) | | | | | *P* for trend ^a^ |
| --- | --- | --- | --- | --- | --- |
|  | Q1  < 53.30 | Q2  53.30-69.22 | Q3  69.22-86.00 | Q4  ≥ 86.00 |  |
| Model 1^b^ | 4.79(3.50, 6.56) | 2.87(2.15, 3.83) | 1.82(1.36, 2.42) | 1.00 | <0 .001 |
| Model 2^c^ | 1.87(1.33, 2.63) | 1.45(1.08, 1.93) | 1.23(0.90, 1.67) | 1.00 | <0 .001 |
| Model 3^d^ | 1.87(1.28, 2.73) | 1.33(0.99, 1.79) | 1.19(0.88, 1.62) | 1.00 | <0 .001 |

^a^ Test for trend based on variable containing median value for each quartile.

^b^ Model 1 was adjusted for Age, Sex, Race/ethnicity, Education status and Family income status.

^c^ Model 2 included model 1 Smoking, Alcohol intake, Physical activity, WC, BMI (Categorical variable: <25, 25-30, ≥30), eGFR.

^d^ Model 3 included model 2 variables plus Hypertension, Diabetes and Hyperlipidemia.
